# Genotypic Responses to Combined Effects of VPD and Salinity in Hydroponically Grown Tomato and Cucumber

**DOI:** 10.1002/pei3.70064

**Published:** 2025-06-06

**Authors:** Hemanth Kumar Puppala, Jörn Germer, Folkard Asch

**Affiliations:** ^1^ Hans‐Ruthenberg‐Institute for Tropical Agricultural Sciences Universität Hohenheim Stuttgart Germany

**Keywords:** growth, leaf area, nutrient uptake, salinity stress, transpiration

## Abstract

To reduce pressure on arable land and water resources, crops can be grown in controlled environments that allow one to recuperate water transpired by plants. This would reduce water demand and potentially allow the use of saline water. However, condensing atmospheric water affects the vapor pressure deficit (VPD), which will affect plant transpiration, nutrient transport, salt uptake, and ultimate growth. This study examined responses of two genotypes of tomato and cucumber during the vegetative phase to varying VPD levels (3.1 and 1.9 kPa) and NaCl concentrations (0 and 30 mM) grown in hydroponic solutions. Under higher VPD (3.1 kPa), transpiration significantly increased in both tomato and cucumber, driving higher water loss. In tomatoes, higher VPD (3.1 kPa) increased the total dry biomass of the Saluoso genotype from 4.3 to 7.1 g and of the Sweeterno genotype from 4.9 to 7.3 g. Root zone salinity diminished the differences in biomass induced by VPD, with little effect on biomass accumulation in both tomato genotypes. Root zone salinity consistently reduced dry weight in cucumber, lowering Addison's from 15.5 to 9.5 g and Proloog's from 13.5 to 10.0 g, regardless of VPD. Unlike tomato, cucumber did not respond to VPD and was more sensitive to salinity. These findings indicate that in hydroponic cultivation, particularly in protected environments, the possibility of producing clean water alongside crop production depends on species‐specific responses. In tomatoes, high VPD enhanced growth and demonstrated compatibility with the use of saline water, supporting the dual goal of productivity and water recovery. However, in cucumbers, the sensitivity to salinity and lack of response to VPD highlight the need for careful species selection and management to achieve sustainable water use and crop production.

## Graphical abstract

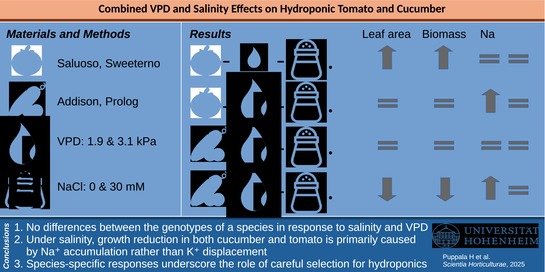

Combined VPD and Salinity Effects on Hydroponic Tomato and Cucumber.

## 1 Introduction

Arable land is increasingly threatened by land‐use change, water scarcity, and degradation through the growing world population and the consequences of climate change (Prăvălie et al. 2021). One way to reduce the pressure on productive land is to cultivate crops highly intensely and efficiently in protected and controlled environments. Plant production in such environments is decoupled from soil, rendering water the most important natural production factor. However, water availability is often limited, and in water‐scarce areas, water sources are often heavily contaminated with salts that are naturally saline (Musie and Gonfa 2023). Thus, protected cultivation depending on limited or marginal water resources needs to grow crops resilient to certain levels of root zone salinity and to be highly water efficient.

The largest water loss in protected cultivation arises from cooling via ventilation, which exhausts the water transpired by the plants during gas exchange (Soussi et al. 2022). Therefore, recovering transpiration water would be an important entry point for increasing the water‐use efficiency in protected cultivation. In closed environments, recovering transpiration water affects air humidity and temperature, and thus vapor pressure deficit (VPD). VPD, the difference between the saturation vapor pressure and the actual vapor pressure of the air, is a crucial factor for plant transpiration, which is the driving force of plant water transport (Yuan et al. 2019) and affects nutrient and salt uptake (Zhang et al. 2021) and thereby significantly affects physiological processes and the growth of plants.

Saline water poses a significant challenge in the protected cultivation of horticultural crops by adversely affecting plant physiological and metabolic processes. High salinity in the rhizosphere leads to osmotic stress, which limits water uptake and ion toxicity, particularly from sodium and chloride, disrupting cellular functions and nutrient balance. These stressors often impair photosynthesis, reduce growth, and ultimately decrease yield and quality of the produce (Niu et al. 2019). In response, plants activate various defense mechanisms, such as ion compartmentalization, osmolyte production, and antioxidant pathways. However, the effectiveness of these responses varies widely among species and cultivars (Zhao et al. 2020).

Hypothetically, in a closed environment, most of the transpiration water could be recovered, thus only the water exported with the yield and surplus biomass would need to be replaced. The recovered water would be fed back into the hydroponic system, whereas the water exported with the biomass and the fruits could be replaced by moderately saline water carrying a salt load lower than the amount exported with the harvest and the surplus biomass. Under such a scenario, the risk of salinization is low, and thus, sustainable crop production with a very high level of water use efficiency could be achieved in water‐scarce and salt‐prone areas.

In this context, it is important to understand how varying VPD conditions affect salt uptake and growth of different species or varieties. Tomato ( *Solanum lycopersicum* L.) and cucumber ( *Cucumis sativus* L.) are economically the most important fruiting vegetables cultivated worldwide (Schreinemachers et al. 2018) with tomato being more salt‐tolerant than cucumber (Alpaslan and Gunes 2001). Adams and Ho (1994) have investigated the combined effects of VPD and salinity on the physiology and growth of cucumber and tomato plants, but the available information on VPD effects remains inconclusive. Therefore, we aim to assess the influence of VPD and salinity on the growth, salt uptake, and water use of two genotypes each of cucumber and tomato.

## 2 Materials and Methods

At the Phytotechnikum, a research greenhouse of the University of Hohenheim, Germany, tomato and cucumber plants were hydroponically grown to study salinity resistance under contrasting VPD levels. Two genotypes of each species were tested: Saluoso and Sweeterno for tomato, and Addison and Proloog for cucumber (seeds provided by Rijk Zwaan Welver GmbH, Welver, Germany). The plants were exposed to two VPD levels (3.1 and 1.9 kPa) and two NaCl concentrations (0 mM and 30 mM) in the hydroponic nutrient solution. Each treatment was replicated three times.

### 2.1 VPD Growth Chambers

Plants were grown in hydroponic systems placed in three separate growth chambers, which served as replicates and were constructed from aluminum frames and transparent acrylic glass. The internal dimensions of each chamber measured 6 × 1 × 1.5 m (L × W × H), and they were divided lengthwise into two compartments. The outer long sides of the chambers were fitted with a plastic curtain, which on the one hand sealed the chamber and on the other allowed access to the experimental setup inside the chamber. In one compartment, vapor flowed from a humidifier (Condair RM, Condair GmbH, Garching‐Hochbrück, Germany). It was evenly distributed by fans, while in the other compartment, the air was circulated through a dryer (CONSORB DC‐10, Seibu Giken DST AB, Spånga, Sweden). Both the humidifier and the dryer were regulated by a hygrostat (HMH, Regin Controls Deutschland GmbH, Berlin, Germany), allowing different humidity levels to be set. The target humidities for the experiment were 80% and 40% during the day phase; the humidity was not regulated during the night phase.

The VPD was calculated by the Tetens' formula (Monteith and Unsworth 2013):

$$\mathrm{VPD}=\mathrm{es}\;\left(T\right)-\mathrm{ea}$$

Where,

$$\mathrm{es}\left(T\right)=0.6108\times \exp\left(\frac{17.27\times T}{T+237.3}\right)$$

T = Air temperature in °C, exp = Exponential function.

$$\mathrm{ea}=\mathrm{es}\left(T\right)\left(\frac{\text{relative humidity}}{100}\right)$$

Artificial light was provided for 14 h a day, from 11:00 a.m. to 1:00 a.m., using 400 W ceramic metal halide lamps, with an intensity of about 450 μmol m^−2^ s^−1^ photosynthetic active radiation at canopy level. The phases of artificial lighting are referred to below as ‘day,’ and the dark phases as ‘night.’

### 2.2 The Hydroponic Systems

**FIGURE 1:**
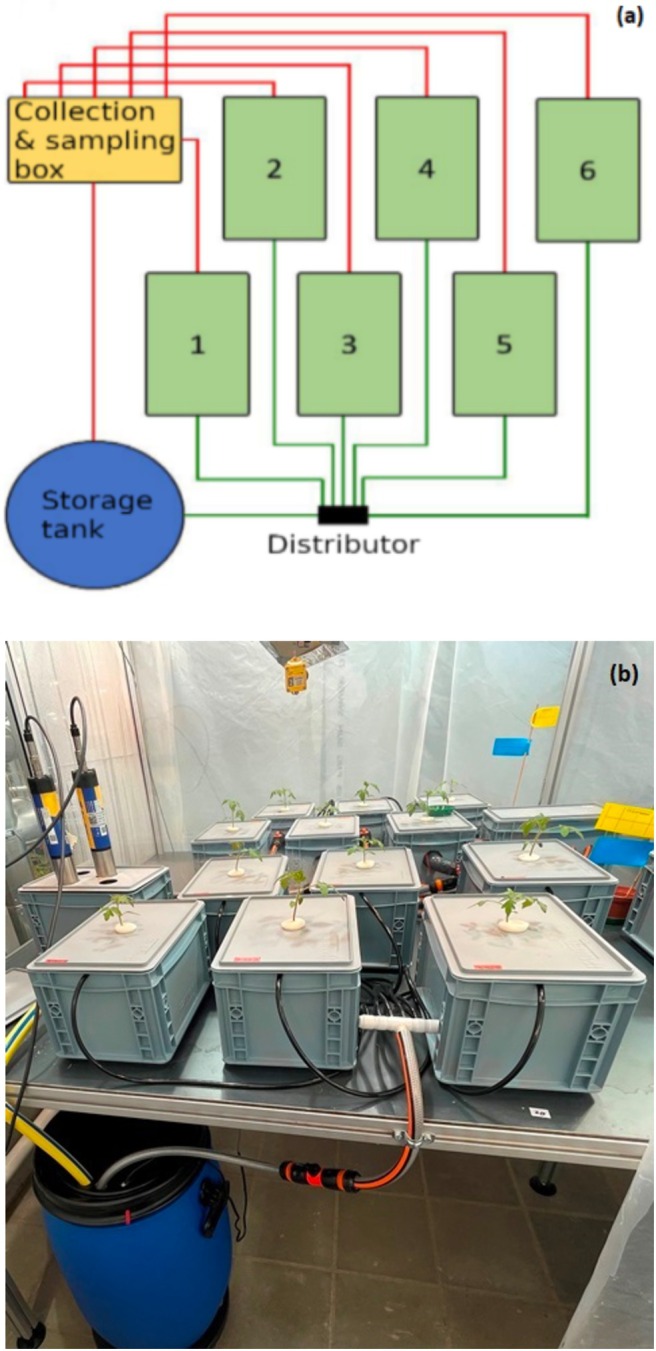
(a) Schematic layout of one hydroponic system with the six plant boxes and (b) a system in operation.

Four hydroponic systems were installed in each compartment of the chambers. Every system consisted of seven boxes: six for harboring plants and one for collecting the return flow from the six plant boxes, which also served as a sampling point (Figure 1). Samples of the nutrient solution were taken from this box every other day. The nutrient solution was pumped by a pump (compactON 1000, EHEIM GmbH & Co. KG, Deizisau, Germany) from a storage barrel through black tubes into the six plant boxes. The solution flowed with a flow rate of 36 L h^−1^ per box through all six boxes, then into the sampling box, and finally back into the barrel. In this way, the nutrient solution, totaling 101.8 L, continuously circulated throughout the system (Figure 1).

### 2.3 Atmospheric Conditions

**FIGURE 2:**
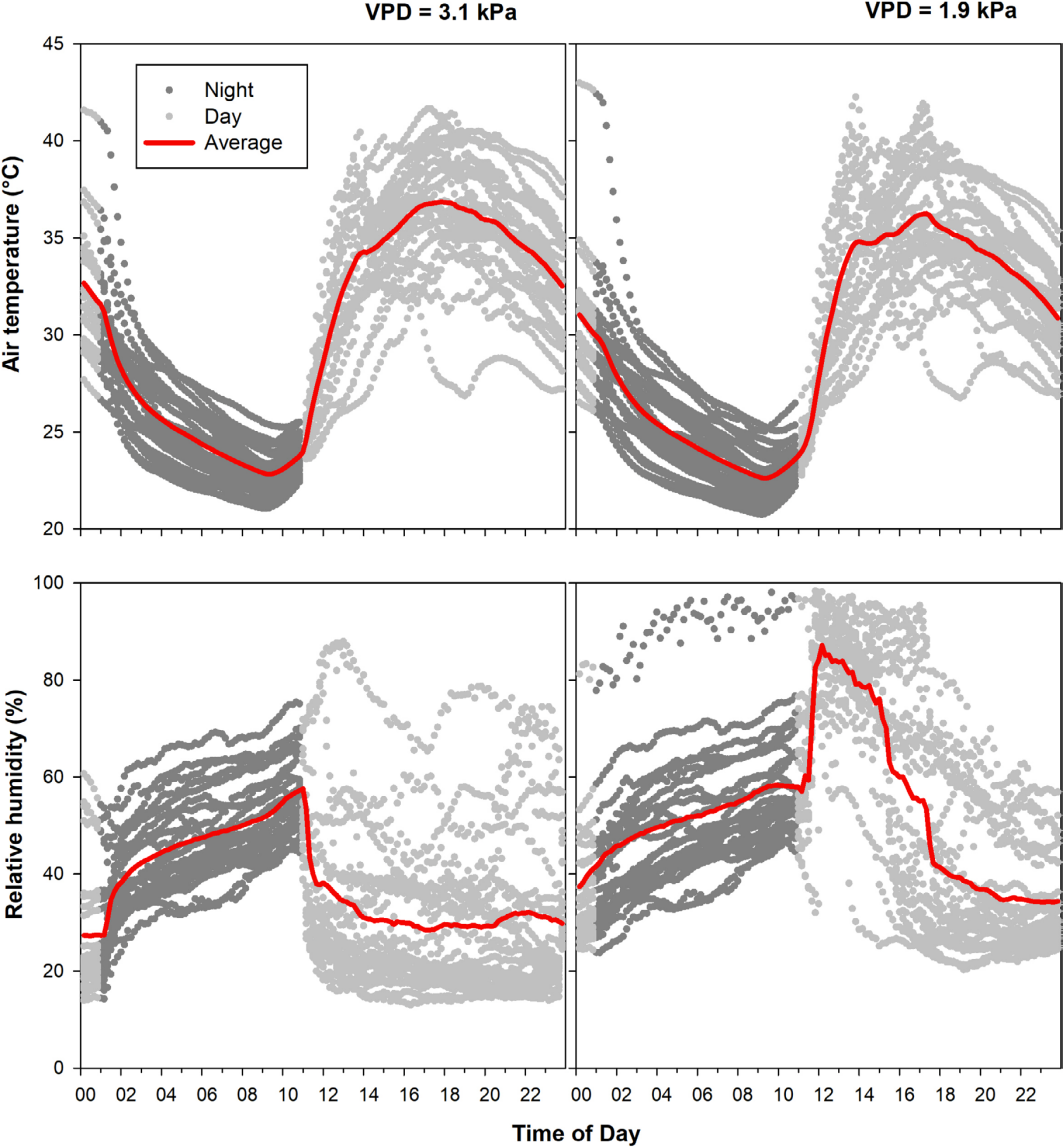
Daily and mean daily air temperature and relative air humidity dynamics in the growth chambers under high VPD conditions (left) and low VPD conditions (right).

The average day and night temperatures were 32.3°C and 27.5°C under high VPD conditions and 31.6°C and 26.9°C under low VPD conditions, while the average VPD during the day was 3.1 ± 1.2 kPa and at night 2.2 ± 0.9 kPa under high VPD and 1.9 ± 1.2 and 2.0 ± 0.6 kPa under low VPD, respectively. Due to the high temperatures in the experimental greenhouse, combined with the additional energy input from the artificial lighting and the operation of humidifiers and dehumidifiers, it was necessary to occasionally suspend humidity regulation to prevent overheating and temperature stress on the plants. As a result, the intended target humidity levels of 40% and 80% could only be maintained to a limited extent (Figure 2).

### 2.4 Nursing, Nutrient Solutions, and Salt Treatments

To improve germination homogeneity, the seeds were treated to 10°C for 48 h before being placed in seedling trays which were filled with sand on June 10, 2022. The seeds germinated into seedlings after 7 days. The seedlings were supplied with a nutrient solution starting from the first day based on a recipe from the Institute of Horticulture Technologies (Integar, https://www.integar.de/), in which the micronutrient content was increased compared to the original formulation of De Kreij et al. (1999). The nutrient solution was prepared from the following compounds: (NH_4_)2H_2_PO_4_, Ca (NO_3_)_2_.4H_2_O, KNO_3_, K_2_SO_4_, KH_2_PO_4_, MgCl_2_.6H_2_O, MgSO_4_.7H_2_O, CaCl_2_.2H_2_O, KCl, C_10_H_12_FeN_2_NaO_8_, MnSO_4_H_2_O, ZnSO_4_.7H_2_O, CuSO_4_.5H_2_O, (NH_4_)6Mo_7_O_24_, and H_3_BO_4_. The final composition of the nutrient solution contained the following concentrations of macro‐nutrients: 40 mg L^−1^ N total (NO_3_^−^‐N + NH_4_^+^‐N), 3.25 mg L^−1^ P, 65.2 mg L^−1^ K^+^, 35.6 mg L^−1^ Ca^2+^, 6.8 mg L^−1^ Mg^2+^, 13.2 mg L^−1^ SO_4_^2−^, 4 mg L^−1^ Cl^−^ and micronutrients: 0.32 mg L^−1^ Fe^2+^, 0.24 mg L^−1^ Mn^2+^, 0.12 mg L^−1^ Zn^2+^, 0.02 mg L^−1^ Cu^2+^, 0.0368 mg L^−1^ B, 0.012 mg L^−1^ Mo. The selected strength of the nutrient solution is based on empirical values in the experimental setup described above.

After four weeks, the seedlings were transplanted into hydroponic systems. All systems were filled with the same solution which was replaced 11 days after the onset of treatments, based on nitrate depletion. The nitrate concentration served as a threshold for the replacement of the nutrient solution; if it fell below 5 mg L^−1^, the nutrient solution was replaced. The pH of the nutrient solution was maintained between 5.5 and 6.0 by the addition of HCl. To set the salt treatment to 30 mM NaCl, 15 mL of 2M NaCl solution was added per liter of nutrient solution immediately after the nutrients were added to the system.

### 2.5 Data Collection and Harvesting Procedure

The electrical conductivity of the nutrient solution was measured, and analysis of NO_3_^−^ in the nutrient solution was performed using an autoanalyzer (Technicon AutoAnalyzer II, SEAL Analytical, New York, USA) daily. Relative humidity and temperature inside the chamber were measured using a Tiny Tag (TGP‐4500, Gemini data loggers, UK). SPAD measurements were taken with a SPAD meter (Minolta SPAD 502, Germany). Daily evapotranspiration was determined by measuring the amount of deionized water required to refill the system to its initial level each day. Cucumber and tomato plants were harvested 24 days after planting. Plants were separated into individual leaves in order of appearance, stems, and root sections. Stem length was measured with a scale, and leaf area was measured with a leaf area meter (Li‐3100, LI‐COR, Lincoln, USA). The separated biomass was individually packed in paper bags and oven‐dried at 65°C for 72 h. The dry weight was determined with a precision balance (XB 220 A, Precisa, Switzerland). SLA and root/shoot ratio were also calculated. Na, Cl, and K contents were analyzed according to Asch et al. (2022). 0.1 g of each finely ground sample was placed into autoclavable tubes and autoclaved at 121°C for 60 min after adding 10 mL of deionized water. The contents of the autoclaved tubes were then filtered into a 100 mL volumetric flask and diluted to 100 mL with deionized water. Subsequently, 20 mL of this solution was transferred into small plastic bottles for analysis using a flame photometer (PFP7, Jenway, UK) for Na^+^ and K^+^ and an autoanalyzer for Cl^−^.

### 2.6 Statistical Analysis

As the focus was on specific conditions rather than the overall interaction among all factors, the data was subjected to a set of two‐way analysis of variance within the defined conditions. Microsoft Excel (Microsoft Corporation, 2024), Sigma Plot 12.5 (Systat Software Inc., San Jose, USA), and the R language (R Foundation for Statistical Computing, 2023) were used to analyze the data.

## 3 Results

### 3.1 Root Zone Salinity and Nutrient Uptake

**FIGURE 3:**
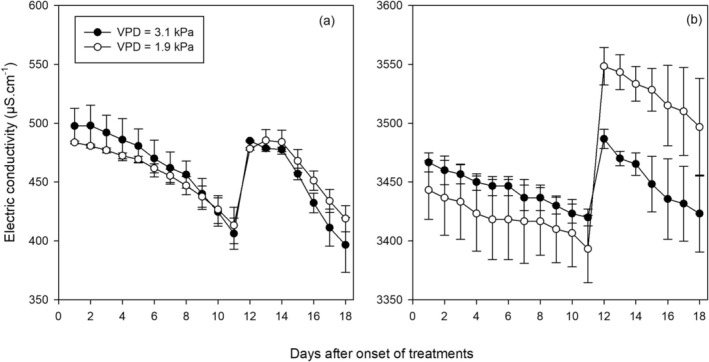
Time course of the electrical conductivity of the (a) non‐saline and (b) saline nutrient solution. Nutrient solutions were changed 11 days after the onset of treatments.

The electrical conductivity (EC) of the nutrient solution (Figure 3) was about 0.5 dS m^−1^ in the non‐saline control treatment. Within 11 days after the onset of the treatments, EC decreased by approximately 0.1 dS m^−1^ in both VPD conditions, indicating an undisturbed uptake of nutrients, particularly NO_3_−. Adding 30 mM NaCl to the nutrient solution increased the EC value by approximately 3.0 dS m^−1^ (Figure 3). Within 11 days after the onset of treatments, EC of the saline treatment was reduced by approximately 0.06 dS m^−1^, indicating a reduced uptake of nutrients from the nutrient solution as compared to non‐saline conditions. Nutrient solutions were changed after 11 days of treatment for all systems. EC values were approximately restored to the initial values and resumed a similar reduction pattern. The slope of the decrease in EC in both saline and non‐saline treatments was steeper in the second phase of the experiment due to the growth of the plants and, thus, an increased requirement for nutrients.

### 3.2 Water Loss and Ion Uptake

**FIGURE 4:**
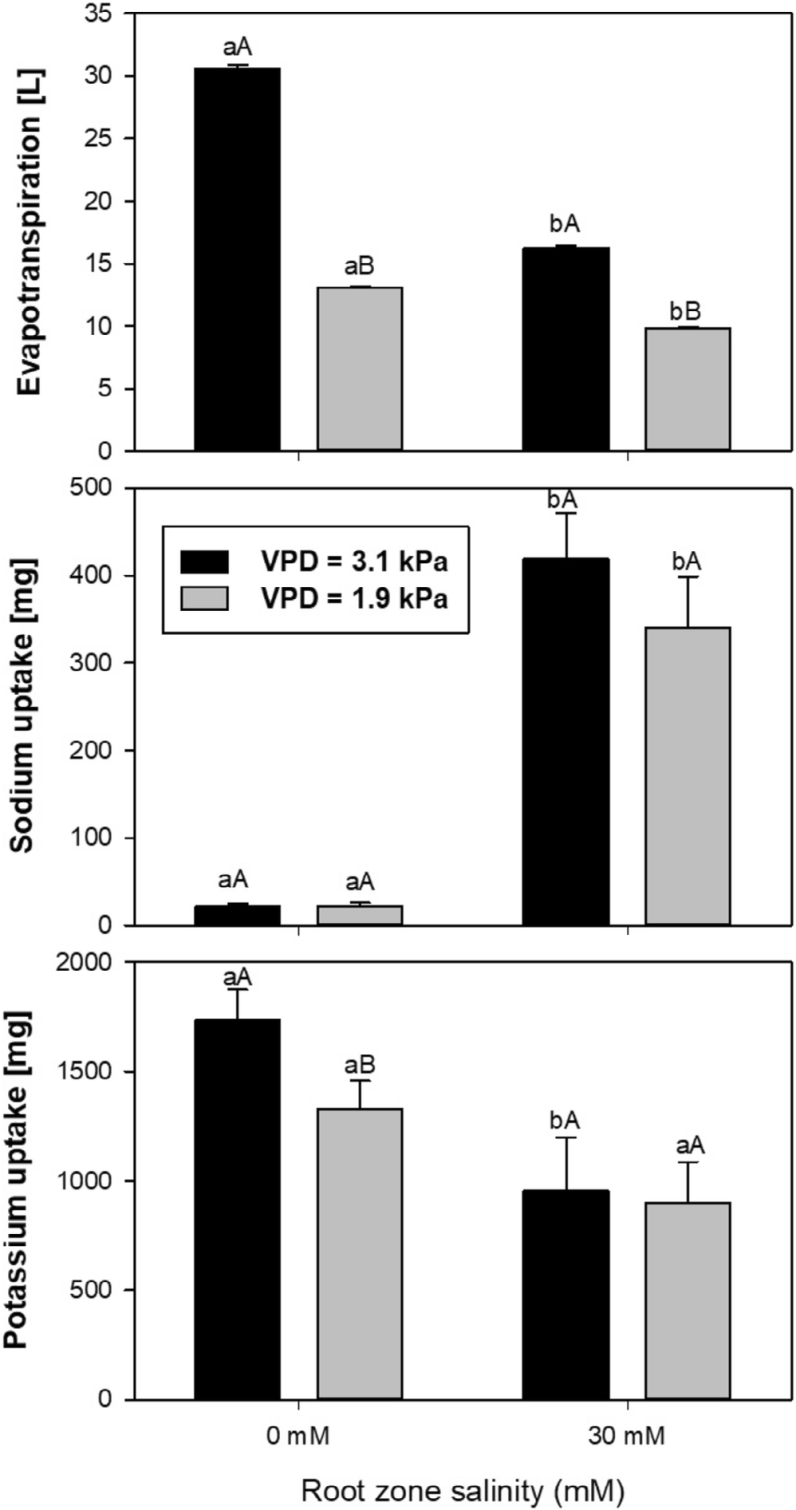
Cumulative water loss via evapotranspiration and the total uptake of sodium and potassium into the biomass per hydroponic set‐up for both VPD environments (3.1 kPa = high VPD; 1.9 kPa = low VPD) and root zone salinity levels (0 mM and 30 mM NaCl). Different letters (a, b) indicate significant differences in evapotranspiration between levels of root zone salinity within the same VPD. Different capital letters (A, B) indicate significant differences in evapotranspiration between VPDs within the same root zone salinity level.

Figure 4 shows the total water loss via evapotranspiration and the total uptake of sodium and potassium into the biomass per hydroponic set‐up for both VPD environments and root zone salinity levels. Compared to low VPD conditions, high VPD significantly increased evapotranspiration from 13 to 31 L (factor 2.4) under non‐saline conditions and from 10 to 17 L (factor 1.7) under 30 mM root zone salinity. Sodium uptake under non‐saline root zone conditions was less than 20 mg over the experimental period, whereas under 30 mM root zone salinity sodium accumulation in the biomass increased to approximately 400 and 350 mg under high and low VPD, respectively, corresponding roughly to 0.6% of the amount present in the root zone. Relating the sodium accumulation to the water loss revealed that the mean concentrations taken up in the two VPD environments correspond to 23.5 mg L^−1^ under high and 35 mg L^−1^ under low VPD conditions, respectively. Compared to non‐saline conditions, potassium accumulation under 30 mM root zone salinity significantly decreased by 43% (from app. 1750 mg under non‐saline conditions to about 1000 mg under saline conditions) under high VPD and 27% (from about 1300 mg to about 950 mg) under low VPD conditions. Root zone salinity thus reduced potassium uptake relative to the amount present in the nutrient solution by 11.26% under high VPD (26.4% was taken up under non‐saline conditions and 15.1% under saline conditions) and 5.28% under low VPD (19.58% was taken up under non‐saline conditions and 14.3% under saline conditions). This indicates a strongly positive effect of high VPD on potassium uptake under non‐saline conditions and a strongly positive effect of low VPD on potassium uptake under saline conditions.

### 3.3 VPD Effects on Growth of Tomato and Cucumber

Tables 1 and 2 show biomass accumulation and partitioning for two varieties of tomato and cucumber grown under two VPD levels. As compared to low VPD, high VPD conditions positively affected biomass accumulation in both tomato varieties (Table 1). High VPD increased total dry weight by a factor of 1.66 and a factor of 1.48 in Saluoso and Sweeterno, respectively. High VPD particularly affected dry matter accumulation of leaves and roots in Saluoso with increases compared to low VPD of factor of 2.0 and 2.1, respectively. This effect was also evident in Sweeterno, but increases were less pronounced with factors of 1.48 and 1.31 for leaves and roots, respectively. This preferential increase in leaf biomass under high VPD conditions resulted in larger leaf areas (factors 1.6 and 1.45 for Saluoso and Sweeterno, respectively) and an increase in leaf thickness by about 16%–17% indicated by the lower SLA in both varieties (Table 1). The latter is also reflected in higher SPAD values indicating no effect of VPD on leaf chlorophyll content.

**TABLE 1:** Effects of high (3.1 kPa) and low (1.9 kPa) VPD on biomass accumulation, biomass partitioning, leaf area, and SPAD in two tomato genotypes (values are means ± standard error, *n* = 3).

	Tomato			
	Saluoso		Sweeterno	
VPD (kPa)	3.1	1.9	3.1	1.9
Total DW (g)	7.09 ± 0.65	4.26 ± 0.32*	7.27 ± 0.62	4.91 ± 0.11*
Leaf DW (g)	4.23 ± 0.42	2.10 ± 0.24*	4.29 ± 0.39	2.35 ± 0.01*
Stem DW (g)	1.89 ± 0.18	1.71 ± 0.13	2.31 ± 0.09	1.88 ± 0.07*
Root DW (g)	0.96 ± 0.04	0.45 ± 0.05*	0.89 ± 0.03	0.68 ± 0.04*
Leaf area (m^2^)	0.17 ± 0.01	0.10 ± 0.01*	0.16 ± 0.01	0.11 ± 0.01*
SLA (m^2^ kg^−1^)	41.84 ± 3.28	49.27 ± 1.80*	39.23 ± 0.83	47.30 ± 3.98*
Stem length (cm)	70.00 ± 0.70	72.00 ± 9.89	77.50 ± 8.28	68.00 ± 8.48
Root/shoot	0.51 ± 0.02	0.26 ± 0.05*	0.38 ± 0.01	0.36 ± 0.01
SPAD	37.63 ± 2.86	34.60 ± 3.43	36.56 ± 2.41	34.90 ± 3.71

* Indicates a significant difference between two VPD levels of that variety (*p* < 0.05); SLA was calculated as the average of individual SLA data points, and the root/shoot ratio is calculated as below‐ground biomass/above‐ground biomass.

**TABLE 2:** Effects of high (3.1 kPa) and low (1.9 kPa) VPD on biomass accumulation, biomass partitioning, leaf area, and SPAD in two cucumber genotypes (values are means ± standard error, *n* = 3).

	Cucumber			
	Addison		Proloog	
VPD (kPa)	3.1	1.9	3.1	1.9
Total DW (g)	15.46 ± 0.99	15.75 ± 0.40	13.80 ± 1.41	13.40 ± 1.45
Leaf DW (g)	11.11 ± 0.82	10.66 ± 0.31	9.24 ± 0.99	8.46 ± 1.08
Stem DW (g)	3.27 ± 0.45	3.41 ± 0.12	3.17 ± 0.23	3.35 ± 0.25
Root DW (g)	1.08 ± 0.13	1.68 ± 0.03*	1.38 ± 0.20	1.61 ± 0.12
Leaf area (m^2^)	0.71 ± 0.07	0.66 ± 0.06	0.54 ± 0.02	0.43 ± 0.05
SLA (m^2^ kg^−1^)	64.03 ± 6.11	64.24 ± 8.23	59.36 ± 6.37	52.09 ± 1.28
Stem length (cm)	97.50 ± 2.47	104.30 ± 9.11	102.60 ± 9.09	115.30 ± 9.62
Root/shoot	0.34 ± 0.09	0.45 ± 0.06	0.43 ± 0.03	0.48 ± 0.01
SPAD	38.60 ± 2.71	31.80 ± 1.74*	41.70 ± 5.23	35.83 ± 0.29*

* Indicates a significant difference between two VPD levels of that variety (*p* < 0.05); SLA was calculated as the average of individual SLA data points, and the root/shoot ratio is calculated as below‐ground biomass/above‐ground biomass.

Biomass accumulation in cucumber was by factors 2.0 and 3.2 greater in tomato under high VPD and low VPD, respectively. In contrast to tomato, VPD had no significant effect on biomass accumulation or partitioning in cucumber (Table 2). In the absence of VPD effects on leaf dry weight and SLA, significantly higher SPAD values in leaves grown under high VPD conditions indicate a better nitrogen acquisition for cucumber under atmospherically dry conditions.

### 3.4 Effects of VPD on the Growth of Tomato and Cucumber Subjected to 30 mM Root Zone Salinity

Tables 3 and 4 show the same parameters as Tables 1 and 2 for plants grown under 30 mM root zone salinity. The shoot biomass of tomato plants did not differ significantly between the non‐saline and salinity conditions. Although in tomato all growth parameters showed a similar trend in response to VPD under saline as under non‐saline conditions, the growth‐promoting effect of high VPD conditions was much smaller (e.g., factor 1.4 and 1.2 in leaves of Saluoso and Sweeterno, respectively) and in most cases not statistically significant under saline root zone conditions (Table 3).

**TABLE 3:** Effects of high (3.1 kPa) and low (1.9 kPa) VPD on biomass accumulation, biomass partitioning, leaf area, and SPAD in two tomato genotypes under 30 mM root zone salinity (values are means ± standard error, *n* = 3).

	Tomato			
	Saluoso		Sweeterno	
VPD (kPa)	3.1	1.9	3.1	1.9
Total DW (g)	6.31 ± 1.06	4.56 ± 1.27	5.61 ± 0.87	5.02 ± 0.79
Leaf DW (g)	3.46 ± 0.71	2.45 ± 0.68	3.20 ± 0.67	2.67 ± 0.52
Stem DW (g)	1.77 ± 0.21	1.44 ± 0.43	1.63 ± 0.10	1.66 ± 0.33
Root DW (g)	1.06 ± 0.13	0.66 ± 0.18*	0.78 ± 0.10	0.68 ± 0.06
Leaf area (m^2^)	0.17 ± 0.01	0.14 ± 0.02	0.14 ± 0.01	0.13 ± 0.01
SLA (m^2^ kg^−1^)	51.52 ± 8.75	65.07 ± 23.55	48.27 ± 9.51	49.60 ± 6.50
Stem length (cm)	65.50 ± 1.76	70.50 ± 2.47	57.00 ± 2.12	69.00 ± 9.79
Root/shoot	0.60 ± 0.01	0.46 ± 0.02*	0.47 ± 0.03	0.44 ± 0.13
SPAD	34.56 ± 1.02	30.90 ± 1.48*	37.20 ± 2.92	30.23 ± 2.10*

* Indicates a significant difference between two VPD levels of that variety (*p* < 0.05), SLA was calculated as the average of individual SLA data points, and the root/shoot ratio is calculated as below‐ground biomass/above‐ground biomass.

**TABLE 4:** Effects of high (3.1 kPa) and low (1.9 kPa) VPD on biomass accumulation, biomass partitioning, leaf area, and SPAD in two cucumber genotypes under 30 mM root zone salinity (values are means ± standard error, *n* = 3).

	Cucumber			
	Addison		Proloog	
VPD (kPa)	3.1	1.9	3.1	1.9
Total DW (g)	9.45 ± 2.30	9.53 ± 2.01	10.32 ± 3.24	9.90 ± 2.20
Leaf DW (g)	6.07 ± 1.67	6.26 ± 1.41	6.70 ± 2.28	6.13 ± 1.46
Stem DW (g)	2.50 ± 0.49	2.42 ± 0.57	2.45 ± 0.68	2.71 ± 0.62
Root DW (g)	0.88 ± 0.18	0.85 ± 0.05	1.16 ± 0.27	1.06 ± 0.14
Leaf area (m^2^)	0.34 ± 0.11	0.38 ± 0.07	0.38 ± 0.15	0.34 ± 0.06
SLA (m^2^ kg^−1^)	56.04 ± 4.05	62.81 ± 3.82	54.54 ± 5.79	56.81 ± 3.64
Stem length (cm)	99.33 ± 14.16	111.00 ± 13.54	96.33 ± 23.24	110.00 ± 14.19
Root/shoot	0.36 ± 0.06	0.38 ± 0.10	0.48 ± 0.05	0.40 ± 0.05
SPAD	31.50 ± 0.85	27.96 ± 0.84*	38.46 ± 1.02	31.16 ± 2.83*

* Indicates a significant difference between two VPD levels of that variety (*p* < 0.05), SLA was calculated as the average of individual SLA data points, and the root/shoot ratio is calculated as below‐ground biomass/above‐ground biomass.

Root zone salinity significantly decreased biomass accumulation in both cucumber genotypes under both VPD levels (Table 4) by about 60% as compared to non‐saline conditions (Table 2). The effect was most pronounced in leaf biomass accumulation, which was reduced by 69% (high VPD) and 77% (low VPD) relative to the non‐saline control in Addison and by 66% (high VPD) and 69% (low VPD) in Proolog.

Whereas root zone salinity did not affect the leaf area of tomato under either VPD level, in cucumber it significantly reduced leaf area by 52% (high VPD) and 42% (low VPD) in Addison and by 30% (high VPD) and 20% (low VPD) in Proolog.

Root zone salinity reduced SPAD values in both genotypes of tomato and cucumber relative to the non‐saline controls. Equally in all genotypes, high VPD resulted in an increase in SPAD values across root zone salinity treatments. In tomato, this increase was always accompanied by a decrease in SLA, whereas in cucumber, SLA in high VPD conditions only decreased under root zone salinity.

### 3.5 Effects of VPD and Salinity on K^+^ and Na^+^ Concentrations in Tomato and Cucumber Leaves, Stems, and Roots

**FIGURE 5:**
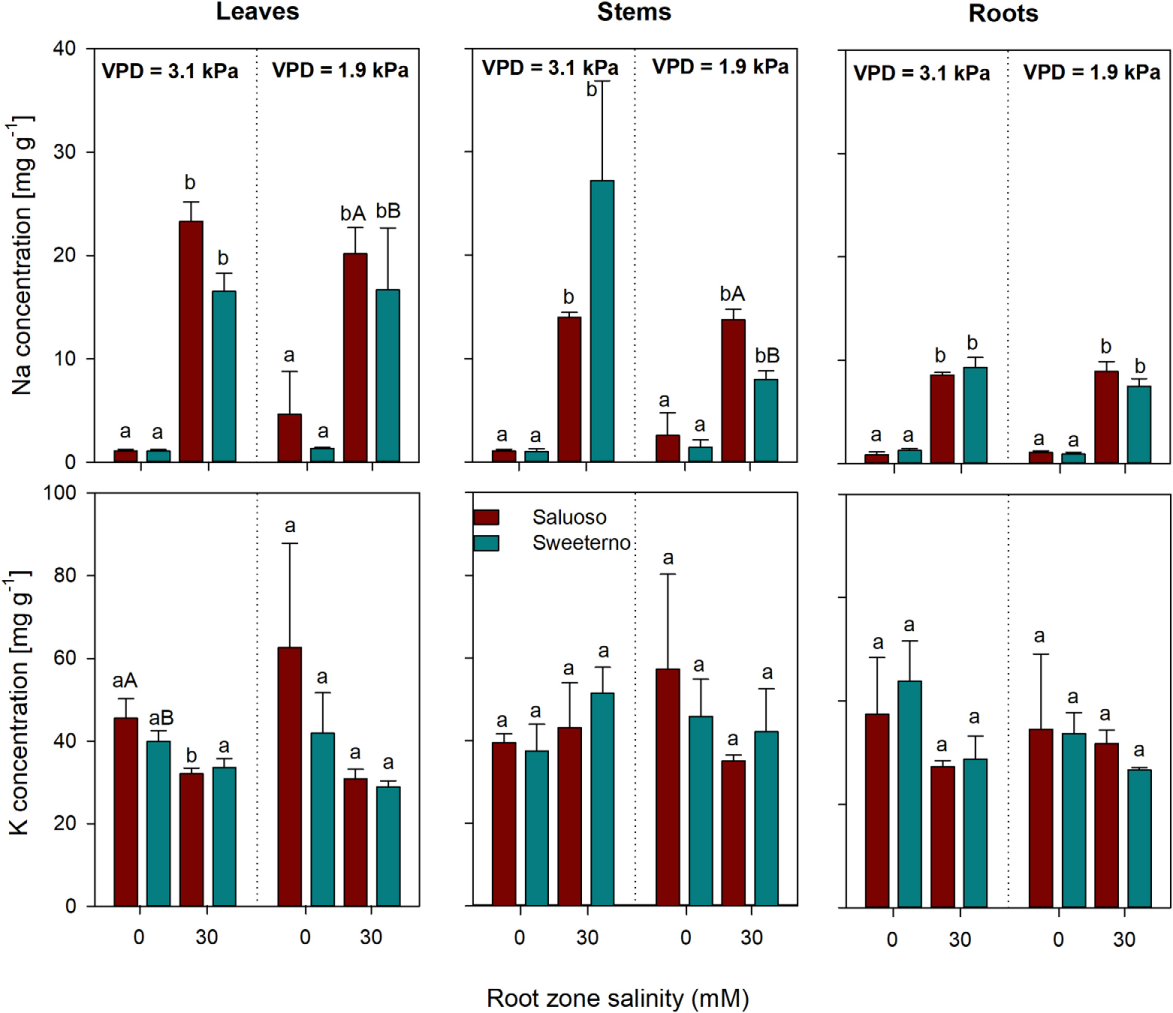
Sodium and potassium concentrations of leaves, stems, and roots of two tomato genotypes (Saluoso and Sweeterno) subjected to 2 levels of root zone salinity (0 and 30 mM of NaCl) and grown under two contrasting vapor pressure deficits (VPD) Different lowercase letters indicate significant differences within a genotype between root zone salinity treatments. Different capital letters indicate significant differences between the two genotypes within the same VPD and salinity level.

**FIGURE 6:**
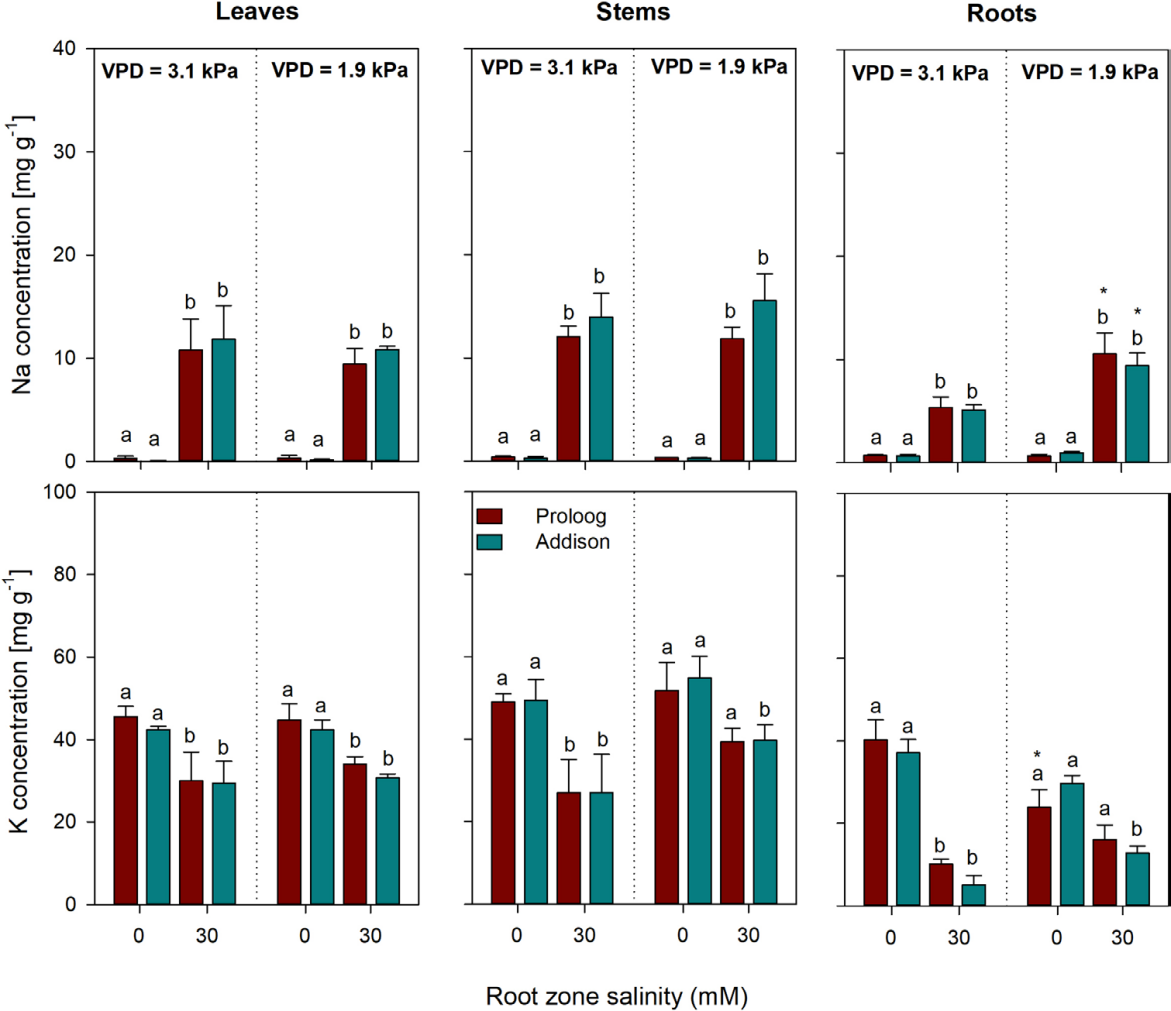
Sodium and potassium concentrations of leaves, stems, and roots of two cucumber genotypes (Proloog and Addison) subjected to 2 levels of root zone salinity (0 and 30 mM of NaCl) and grown under 2 contrasting vapor pressure deficits (VPD) Data are presented as means ± standard error, *n* = 3. Different lowercase letters indicate significant differences within a genotype between root zone salinity treatments. Different capital letters indicate significant differences between the two genotypes within the same VPD and salinity level.

Figures 5 and 6 show mean sodium and potassium concentrations for leaves, stems, and roots for two tomato varieties and two cucumber varieties grown under two contrasting VPD conditions subjected to two root zone salinity levels. In general, salinity significantly increased the sodium concentration in all organs and genotypes, but no additional effects were observed for differences in VPD (Figures 5 and 6). Sodium accumulation was stronger in tomato (10 and 25 mg g^−1^, Figure 5) than in cucumber (10 and 15 mg g^−1^, Figure 6). Whereas no significant differences in organ sodium concentrations were observed in cucumber (Figure 6), leaf and stem sodium concentrations in the two tomato varieties differed more strongly (Figure 5), particularly under low VPD.

Potassium concentration in the different organs tended to be higher under non‐saline conditions; however, potassium concentrations in leaves, stems, and roots were only significantly higher in cucumber under non‐saline conditions regardless of the VPD environment (Figure 6). In tomato, neither root zone salinity nor VPD had any significant effect on potassium concentrations in the respective organs.

### 3.6 Effects of Salinity on K^+^ and Na^+^ Concentrations in Tomato and Cucumber Grown Under High and Low VPD

**FIGURE 7:**
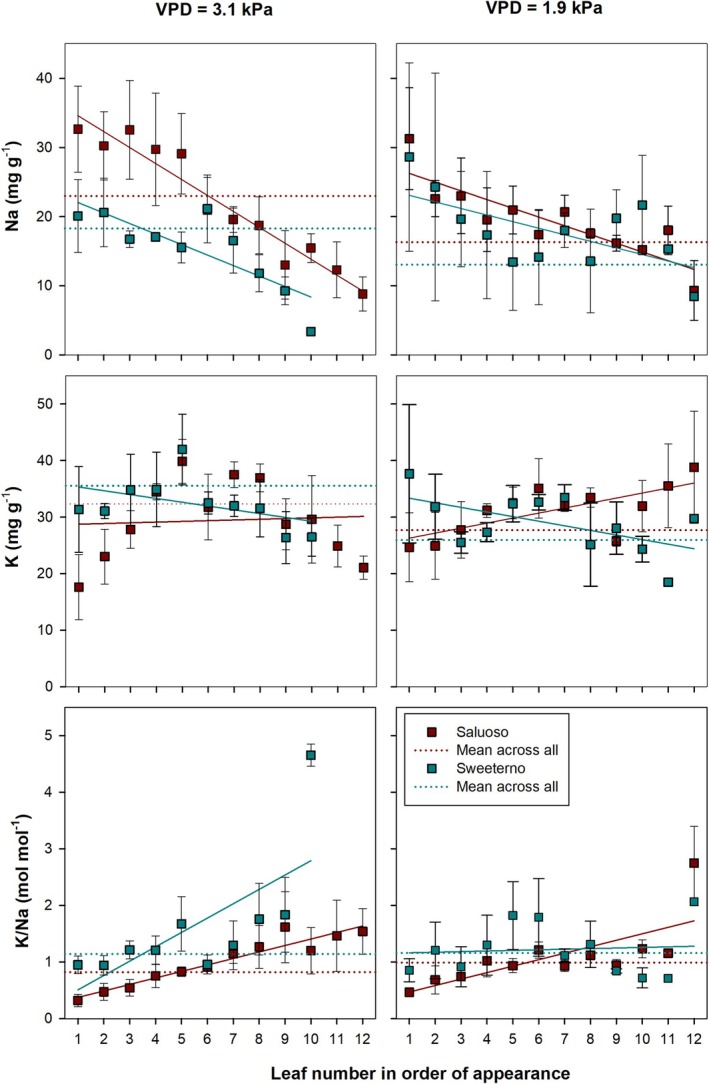
Sodium and potassium concentrations and the resulting molar K/Na ratio for leaves in order of appearance of two tomato genotypes (Saluoso and Sweeterno) grown under 30 mM root zone salinity subjected to high (3.1 kPa) and low (1.9 kPa) VPD. Solid lines represent linear regressions of the presented parameter against leaf age. Dotted lines represent the average across all leaf positions. Data are presented as means ± standard error, *n* = 3.

**FIGURE 8:**
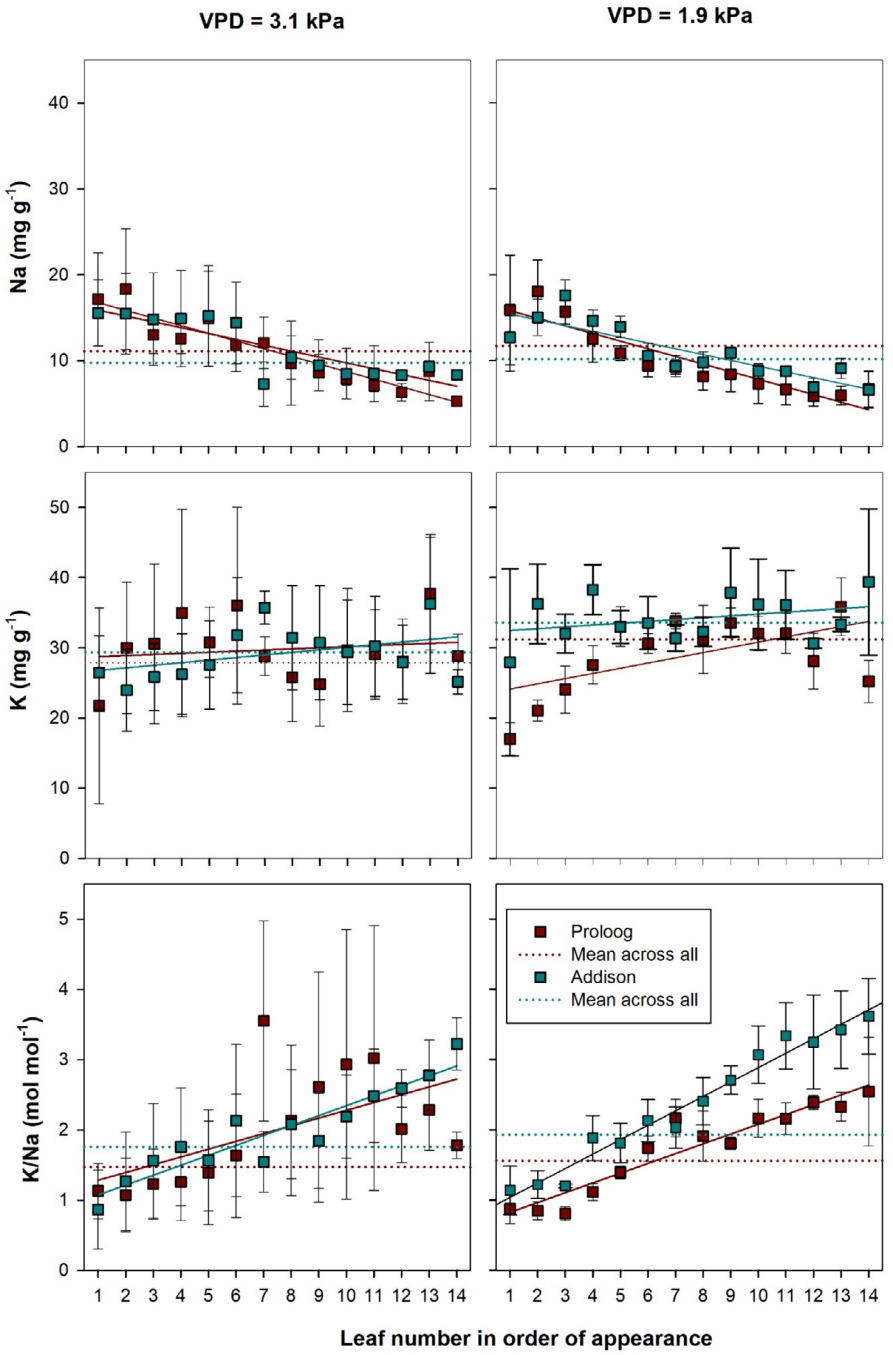
Sodium and potassium concentrations and the resulting molar K/Na ratio for leaves in order of appearance of two cucumber genotypes (Proloog and Addison) grown under 30 mM root zone salinity subjected to high (3.1 kPa) and low (1.9 kPa) VPD. Solid lines represent linear regressions of the presented parameter against leaf age. Dotted lines represent the average across all leaf positions. Data are presented as means ± standard error, *n* = 3.

We measured the individual concentrations of Na^+^ and K^+^ in all leaves in order of their appearance in all cultivars of tomato and cucumber for all treatments. As seen in Figures 5 and 6, Na accumulation under non‐saline root zone conditions was very low in all organs; thus, we report here only the results obtained under 30 mM root zone salinity. Figure 7 (tomato) and Figure 8 (cucumber) depict the sodium and potassium concentration and the resulting molar K^+^/Na^+^ ratio of 12 and 14 successive leaves for tomato and cucumber, respectively, for high and low VPD conditions.

In tomato (Figure 7), leaf sodium concentration was negatively linearly correlated with leaf age in both varieties and under both VPD conditions. Under high VPD, with each leaf position, sodium concentration significantly decreased by 2.3 mg (*r* = 0.97, slope −2.30) in Saluoso, whereas in Sweeterno, sodium decreased by 1.52 mg per leaf position (*r* = 0.82, slope −1.52). Similar trends were found under conditions of low VPD, but the decrease per leaf position was smaller by 45% in Saluoso and by 38% in Sweeterno (*r* = 0.86, slope −1.26 and *r* = 0.63, slope −0.95, respectively). For potassium, no linear relationships between concentration and leaf position were found. Leaf K concentrations varied between 18 and 42 mg g^−1^, independent of variety and VPD. In both varieties and under both VPD conditions, the middle leaves (positions 4–8) showed the highest and above‐average potassium concentrations. The apparent negative (Sweeterno) and positive (Saluoso) correlations under low VPD conditions were not statistically significant (*r* = 0.56 and *r* = 0.7, respectively). The resulting K^+^/Na^+^ ratio was calculated on a molar basis for the respective leaf concentrations. In Saluoso, the K^+^/Na^+^ ratio was significantly positively correlated with leaf position, with an almost parallel slope for VPD conditions (*r* = 0. 95, slope 0.12—high VPD; *r* = 0.74, slope 0.11—low VPD) In Sweeterno, only under high VPD conditions was a positive correlation found between leaf K^+^/Na^+^ ratio and leaf position; however, this correlation depended strongly on the high K^+^/Na^+^ ratio in the youngest leaf and was not statistically significant.

In cucumber (Figure 8), leaf sodium concentration showed a similar trend as in tomato, but the individual concentrations stayed in a narrower range between 6 and 19 mg g^−1^ as compared to 9 to 35 mg g^−1^ in tomato. Neither VPD nor genotype effects were observed. Leaf sodium concentration decreased significantly per leaf position by about 0.89 mg g^−1^ in Proloog (*r* = 0.93) and about 0.68 mg g^−1^ in Addison (*r* = 0.86) independent of the VPD environment. Leaf potassium concentrations varied across the leaf positions between 18 and 35 mg g^−1^ in all varieties and for all VPD environments with no correlation between the potassium concentration of the leaves and their age. Leaf K^+^/Na^+^ ratios varied widely, particularly for leaf positions 6–11 under high VPD conditions, which reflects the variability in leaf potassium concentrations. The trend toward higher K^+^/Na^+^ ratios in younger leaves was more pronounced in cucumber than in tomato, with a significantly positive correlation between leaf position and K^+^/Na^+^ ratio in the genotype Addison (*r* = 0.93, slope 0.14) under high VPD conditions. Under low VPD conditions, this significant correlation was preserved; however, the genotypes differed strongly in slope (Addison: *r* = 0.98, slope 0.2; Proloog: *r* = 0.89, slope 0.14).

## 4 Discussion

Several factors need to go hand in hand to render plant production in controlled environments resource‐efficient, water‐saving, and sustainable. As in all agricultural systems, energy use, water use, land use, and nutrient use should be minimal, balanced, and, whenever possible, circular (Asch and Huelsebusch 2009). In the case of water use in hydroponically grown plants, the use of marginal waters (saline surface or ground waters or domestic wastewater) in plant production could take some pressure off freshwater resources while increasing the productivity of the hydroponic system. For this to function, plants must be able to grow in the nutrient solutions provided. If salinity cannot be avoided, a suitable crop needs to be selected and the crop threshold as well as salinity effects on the crop growth, and the capacity of the crop to take up sodium into its biomass for potential removal from the system, need to be known. Environmental conditions such as air humidity and temperature affect photosynthesis and transpiration via the atmospheric VPD and, thus, nutrient uptake and growth are key to successfully growing plants under controlled environmental conditions. Many published studies investigate VPD effects outside of the typical range found in commercial greenhouses. Table 5 presents a comprehensive overview of VPD conditions used in various studies on vegetable crops, some examples of conditions in greenhouses used for tomato production, and the conditions used in this study.

**TABLE 5:** Type of controlled environment and definitions of low and high VPD used in several scientific and production‐oriented publications on vegetable crops.

Environment	Low VPD (kPa)	High VPD (kPa)	Crop	Purpose of the study	References
***rH‐controlled greenhouse***	***1.9 ± 1.2***	***3.1 ± 1.2***	***Tomato, cucumber***	***Science***	***This study***
semi‐controlled Venlo‐type greenhouse	0.25	1.01	Tomato	Science	Barker (1990)
environmentally controlled growth chambers	0.23	1.05	Faba bean	Science	Aliniaeifard et al. (2014)
Polyethylene Film‐covered greenhouse no ventilation	0.64	13.6	No crop	Production/Science	Shamshiri et al. (2014)
Polycarbonate‐covered greenhouse, evaporative cooling	0.21	3.97	No crop	Production/Science	Shamshiri et al. (2014)
Climate‐controlled greenhouse	< 1.5	3.2–4.2	Muskmelon, cucumber	Science	Song et al. (2021)
environmentally controlled growth rooms	0.63	1.67	Tomato	Science	Zhang et al. (2021)
environmentally controlled growth rooms	0.9	2.2	Tomato	Science	Ding et al. (2022)
semi‐controlled Venlo‐type greenhouse	4–6	7–10	Cucumber	Production/Science	Luo et al. (2005)
semi‐closed greenhouse, horizontal ventilation	0.9	2.8	Tomato	Production/Science	Jerszurki et al. (2021)

Most scientific studies employ VPD environments considerably lower than what can be expected in a production‐oriented environment (Table 5). Thus, the transferability of such results into practical application may be limited. We presented here data on growth, salt uptake, and transpiration of two varieties each of tomato and cucumber hydroponically grown under controlled environmental conditions subjected to two levels of root zone salinity and two levels of realistic VPD. We used environmental conditions as close as possible to those expected in subtropical greenhouse production systems.

### 4.1 VPD Effects on Biomass Accumulation of Tomato and Cucumber

In tomato, high VPD under non‐saline conditions significantly increased dry matter accumulation, particularly in leaves and roots (Table 1). Since the increase in leaf area was less pronounced, the leaf mass per unit area increased, indicating an increase in leaf thickness. Thicker leaves resulted in higher chlorophyll density, as evidenced by the increased SPAD values, indicating a higher CO_2_ assimilation rate. Barker (1990) reported that under low VPD and non‐saline conditions, less calcium is transported to young expanding leaves, thus reducing leaf area expansion, whereas high VPD promotes leaf area expansion and enhances the transpiration rate of tomato plants, thereby promoting nutrient transport. In combination, these effects resulted in significant differences in biomass as compared to low VPD conditions.

These findings contradict more recent reports (Zhang et al. 2021; Ding et al. 2022) where high VPD was found to limit plant biomass accumulation in tomato. These contrasting observations are likely due to differences in VPD patterns. Zhang et al. (2021) as well as Ding et al. (2022) subjected their tomato varieties to VPD levels that were significantly more humid than those in the present study and were, in contrast to this study, kept constant day and night. Barker (1990) has shown that constant, long‐term high humidity is detrimental to tomato growth and fruit quality, whereas Leuschner (2002) showed that plants ecologically adapted to high humidity conditions thrive better under low VPD conditions than under high VPD conditions. In contrast, in faba bean, sustained exposure to low VPD reduced leaf capacity to maintain adequate water status (Aliniaeifard et al. 2014).

Under low VPD conditions, limited transpiration caused an imbalance between water uptake and loss, leading to increased water pressure in the leaves. This pressure build‐up eventually caused cell enlargement and eruption. Blisters developed on the lower surface of the leaves, and on the upper surface of the leaves, brown necrotic spots surrounded by chlorosis (oedema) first documented in detail by Atkinson (1893) and recently illustrated by Kovach and Mattson (2019) and Scheckelhoff (2016). The damage reduced leaf area and decreased transpiration and photosynthesis, which contributed further to reduced biomass accumulation. As the temperature in the growth chambers reached well above 30°C, we assume that the stomata were fully opened (Kostaki et al. 2020) and conclude that transpiration in tomato was limited by the stomatal conductance potential of the leaves under humid conditions. Therefore, in tomato production, as observed in lettuce (Inoue et al. 2021), fine regulation of VPD seems to be important to realize the benefits of high VPD conditions on plant growth. In contrast to tomato, high VPD had no positive effect on cucumber (Table 2), which supports the results of Song et al. (2021), who reported negative effects on the growth and yield of cucumber grown under similar high VPD conditions as in this study. Tomato benefitting from high VPD may have been a consequence of increased transpiration and probably increased stomatal density fostering coordinated regulation of both stomatal and root development to balance water uptake and loss, as observed earlier in hydroponically grown *Arabidopsis* (Hepworth et al. 2016).

### 4.2 Biomass Accumulation of Tomato and Cucumber Under NaCl Salinity

Tomato is considered moderately tolerant to salinity (Guo et al. 2022), but its actual tolerance varies widely based on several factors, including genotype, growth stage, environmental conditions, and salt composition. Under NaCl‐induced salt stress, these factors significantly influence the tomato plant's growth, biomass accumulation, and overall productivity. Accordingly, recommendations on the nutrient solution EC vary widely. Some researchers suggest a range between 1.2 and 1.4 dS m^−1^ (Nasir and Sato 2023). Others report reduced photosynthetic activity above 2.3 dS m^−1^ (Del Amor et al. 2001) while depending on the genotype, the highest threshold can be expected to range between 4 and 6 (Cuartero and Fernández‐Muñoz 1998). Therefore, the EC above 3 dS m^−1^ in our experiment might have already passed the salinity threshold of the used varieties, but not strong or long enough to result in significant biomass reduction or SPAD alteration. This assumption is supported by the higher standard error of all measured parameters (Tables 1 and 3). In general, there are few studies investigating the effect of salt on tomato plants in hydroponic systems, and hardly any studies focus specifically on substrate‐free hydroponic systems. Understanding how salt tolerance varies across different crops and conditions could provide valuable insights for optimizing hydroponic tomato cultivation under saline environments. For example, in rice, sensitive genotypes are susceptible to damage even at low salinity levels of 2 dS m^−1^ (Maas and Hoffman 1977). Asch and Wopereis (2001) found that rice yields can decrease by 50% when exposed to moderate salinity levels of 3.5 dS m^−1^. In contrast, sweet potato exhibits a higher threshold for salinity damage at 75 mM root zone salinity (Mondal et al. 2022). Tanji and Kielen (2002) noted that moderately tolerant crops, such as wheat, barley, or maize, can withstand irrigation water salinity levels around 7 dS m^−1^, while more sensitive crops, such as rice, beans, sugar cane, or apple, are limited to approximately 4.5 dS m^−1^. In Jatropha, the salinity response threshold is within a range of 0–5 dS m^−1^ (Rajaona et al. 2012), highlighting the diversity in salinity tolerance across crop species and genotypes.

Apart from a higher root mass in Saluoso and higher SPAD values in both varieties at high VPD, the plants showed no significant differences between the VPD values under 30 mM NaCl exposure. We noted an increase in the root‐to‐shoot ratio in both Saluoso and Sweeterno, which was significant only under high VPD. However, no such increase was observed in the cucumber genotypes. The impact of salinity on plants is generally moderated under low VPD (Papadopoulos and Rendig 1983). This effect was likely partially offset by the high ambient temperatures in our experiment. High VPD reduces biomass and leaf area in tomatoes, but this effect diminishes with a root zone salinity of 30 mM and is expected to vanish as salinity approaches its threshold. Under non‐saline conditions, hydroponically grown tomatoes typically experience a relatively dry shoot environment. Additionally, since salt stress generally reduces biomass formation across crops, maintaining low salinity is beneficial. However, in the case of tomatoes, if the goal is to recover transpiration water, high VPD is not particularly detrimental when plants are grown under moderate root zone salinity, and transpiration rates remain high under high VPD conditions.

According to Osawa (1965), the salinity threshold of cucumber was 2.5 dS m^−1^. In contrast, the induced salinity level of 30 mM NaCl in our experiment significantly reduced cucumber plant biomass. This suggests that the threshold for the cucumber varieties we tested may have been reached even with a lower NaCl concentration.

The decrease in growth observed at salinity can be attributed to several factors, but in our case ion toxicity, caused by excessive uptake of Na^+^, probably played the most important role (Chen et al. 2020). Like in tomato, the effect of salinity on cucumber is influenced by the specific salt composition. Research indicates that cucumber exhibits a greater sensitivity to NaCl compared to CaCl_2_ (Trajkova et al. 2006). Importantly, chloride ions have minimal direct effects on plant physiology, whereas sodium ions contribute primarily to stress and adverse effects in the plants. Consequently, data on chloride concentrations have been placed in the Appendix for reference. Further, the EC of 3.5 dS m^−1^ was not much higher than common recommendations for cucumber nutrient solution of around 2.7 dS m^−1^. Even at values of up to 3.6 dS m,^−1^ no reduction in leaves and fruits must be anticipated (Shrestha et al. 2024).

### 4.3 Effects of VPD on Leaf K^+^ and Na^+^ Concentrations in Tomato and Cucumber Subjected to Root Zone Salinity

As anticipated, adding salt to the nutrient solution significantly increased Na^+^ concentrations in the analyzed organs of both cucumber and tomato. Alongside this increase in Na^+^, a minor reduction in K^+^ levels was observed in certain cucumber leaves and stems (Appendix 1). Unlike previous studies, we did not observe a strong negative correlation between Na^+^ and K^+^ concentrations in the leaves. For instance, Giuffrida et al. (2009) reported a consistent rise in Na^+^ levels and a decline in K^+^ across five levels of salinity in tomato leaves, ranging from 7 to 64 mM NaCl. Like in our results, the authors also noted that Na^+^ concentrations increased with leaf age.

The observed decrease in biomass accumulation and the high standard error in dry weights across plant organs suggest that the salinity level of 30 mmol may approach the tolerance threshold for both tomato genotypes. The Na^+^ to Cl^−^ ratio in cucumber leaves, about 0.5 mmol/g^−1^, closely aligns with findings by Chen et al. (2020), who concluded that cucumber plants struggle to limit Na^+^ accumulation in their leaves. This excessive Na^+^ buildup is likely responsible for the observed reduction in cucumber biomass under salt stress, rather than the small, non‐significant reduction in K^+^. The constant K^+^ content observed between high and low VPD conditions suggests no disruption to stomatal function or gas exchange, further supporting the notion that Na^+^ toxicity, rather than K^+^ displacement, may explain the reduction in yield.

In agreement with other research (Cuartero and Fernández‐Muñoz 1998), we observed that Na^+^ accumulation in leaves increased with leaf age in both tomato and cucumber, but this effect was more pronounced under high VPD conditions. Given that water loss through transpiration is closely tied to VPD (Mondal et al. 2024; Grossiord et al. 2020), the greater transpiration rate likely contributed to the increased Na^+^ concentrations in older leaves under high VPD. Although we could not precisely measure transpiration rates due to the experimental setup (Figure 3), it can be inferred that transpiration was significantly higher under high VPD than under low VPD. This finding contrasts with previous observations in sweet potato ( *Ipomoea batatas* L.) genotypes, where ion distribution under salt stress was not linked to transpiration (Mondal et al. 2024), indicating that species respond differently to salinity and VPD stress, as seen in our study.

Tomato plants exhibited a steep Na^+^ gradient from older to younger leaves under high VPD, while cucumber showed the opposite trend, highlighting species‐specific responses to salinity and VPD. Furthermore, tomato genotypes responded differently to NaCl stress under varying VPDs, as also reported by Mondal et al. (2024) who observed that salt‐tolerant sweet potato genotypes accumulated significantly more Na^+^ and K^+^ in their petioles than in leaf blades. In our study, although not statistically significant, the tomato genotype Sweeterno exhibited higher Na^+^ concentrations under high VPD compared to Saluoso, suggesting that Sweeterno may utilize its stem to sequester Na^+^ and protect its leaves, a hypothesis that warrants further investigation.

The SPAD values indicated increased chlorophyll content in tomato leaves under high VPD with salt addition, though NaCl alone did not affect SPAD values. Other studies suggest that significant reductions in SPAD occur only above an EC of 6.0 dS m^−1^ (Al‐Gaadi et al. 2024). This indicates that NaCl may have further impeded transpiration under low VPD (Baba and Fujiyama 2003), whereas high VPD's promoting effect on transpiration likely compensated for this impact.

Overall, cucumber and tomato exhibited different responses to the combined effects of VPD and salinity. High VPD promoted tomato growth in non‐saline conditions, while low VPD mitigated the negative effects of salinity. Conversely, VPD had no significant effect on cucumber growth, though salinity substantially reduced its biomass. These results highlight the species‐specific nature of VPD‐salinity interactions and the importance of genotype selection in managing salt stress. Although the differences in biomass reduction between the tomato variety Sweeterno and the cucumber variety Addison were not statistically significant, prior research has demonstrated notable genotypic variation in salt tolerance (Dasgan et al. 2002; Zhu et al. 2008).

## 5 Conclusions

This study examined the effects of VPD on growth, transpiration, and ion distribution in cucumber and tomato genotypes under saline and non‐saline conditions. In tomatoes, higher VPD increased transpiration and biomass under non‐saline conditions, particularly in the Sweeterno genotype. However, salinity reduced biomass due to Na^+^ accumulation in older leaves, with VPD having a less pronounced impact. Tomatoes maintained stable K^+^ concentrations, suggesting Na^+^ toxicity was the primary growth limitation, with genotype‐specific mechanisms like Na^+^ sequestration in stems evident. In contrast, cucumbers showed stable transpiration rates across VPD levels but were highly sensitive to salinity, suffering significant biomass reductions due to Na^+^ accumulation. These results suggest tomatoes are better suited for hydroponic systems that recover transpired water, even under saline conditions, while cucumbers are more vulnerable to salinity stress. Future research should optimize hydroponic strategies for tomatoes to maximize resource use efficiency and yield while also improving salt tolerance in sensitive crops such as cucumber to enable sustainable production in saline environments. Additionally, building on the understanding of genotypic interactions with environmental factors during the vegetative phase, the next step should involve evaluating productivity under similar conditions, focusing on combined genotypic effects and environmental factors. This will require a new experimental setup and approach, particularly for the stages leading to fruit harvest, to better understand their impact on overall yield.

## Conflicts of Interest

The authors declare no conflicts of interest.

## Data Availability

The data that support the findings of this study are openly available in All Data at https://bwsyncandshare.kit.edu/apps/files/files/4632757639?dir=/&openfile=true.

## Appendix 1 Plant Organ‐Specific Ion Concentrations (mg g^−1^) for Both Genotypes of Tomato and Cucumber Subjected to Two Salinity Treatments

**Table:**

Sp	V	VPD	T	Root			Stem			Leaf		
				Na	K	Cl	Na	K	Cl	Na	K	Cl
1	1	1.9	0	1.1 ± 0.1	17.2 ± 7.3	3.3 ± 0.8	2.6 ± 2.2	57.2 ± 22.3	12.5 ± 5.2	4.6 ± 1.4	62.7 ± 25.1	9.4 ± 2.9
1	1	1.9	30	8.9 ± 0.9	15.9 ± 1.3	16.3 ± 0.9	13.8 ± 1.0	35.0 ± 1.5	28.4 ± 3.1	20.2 ± 16.7	30.9 ± 2.2	48.6 ± 1.7
1	1	3.1	0	0.8 ± 0.3	18.7 ± 5.5	4.2 ± 1.2	1.1 ± 0.2	39.5 ± 2.2	7.9 ± 0.2	1.1 ± 0.1	45.5 ± 4.7	5.5 ± 0.8
1	1	3.1	30	8.6 ± 0.3	13.6 ± 0.6	14.8 ± 0.7	14.0 ± 0.5	43.1 ± 10.8	32.6 ± 2.4	23.3 ± 1.9	32.1 ± 1.4	55.5 ± 3.27
1	2	1.9	0	0.9 ± 0.2	16.9 ± 2.0	3.1 ± 0.2	1.4 ± 0.7	45.9 ± 9.0	11.4 ± 3.9	1.4 ± 0.1	41.9 ± 9.7	9.6 ± 1.7
1	2	1.9	30	7.5 ± 0.7	13.3 ± 0.2	13.1 ± 0.7	7.9 ± 0.8	42.1 ± 10.4	33.8 ± 6.1	16.7 ± 6.0	28.9 ± 1.5	41.9 ± 1.0
1	2	3.1	0	1.3 ± 0.2	21.9 ± 3.9	4.9 ± 0.7	1.0 ± 0.3	37.4 ± 6.5	8.8 ± 1.2	1.1 ± 0.2	39.8 ± 2.6	10.1 ± 0.9
1	2	3.1	30	9.3 ± 0.9	14.4 ± 2.2	15.6 ± 1.8	27.2 ± 9.6	51.5 ± 6.3	68.5 ± 19.7	16.5 ± 1.7	33.5 ± 2.2	58.9 ± 12.3
2	3	1.9	0	0.7 ± 0.1	23.9 ± 4.3	3.8 ± 2.1	0.3 ± 0.1	51.7 ± 3.3	3.4 ± 0.8	0.3 ± 0.2	44.7 ± 3.9	4.4 ± 1.0
2	3	1.9	30	10.6 ± 2.0	16.0 ± 3.6	20.7 ± 2.4	11.8 ± 1.1	39.4 ± 6.9	26.6 ± 4.3	9.4 ± 1.5	34.1 ± 1.8	24.5 ± 2.6
2	3	3.1	0	0.7 ± 0.1	40.3 ± 4.8	3.7 ± 2.3	0.4 ± 0.1	49.1 ± 1.9	5.2 ± 1.3	0.3 ± 0.2	45.5 ± 2.5	2.5 ± 0.2
2	3	3.1	30	5.4 ± 1.0	10.1 ± 1.2	14.7 ± 3.6	12.1 ± 1.0	27.1 ± 7.9	22.2 ± 1.0	10.8 ± 2.9	29.9 ± 6.9	22.4 ± 1.3
2	4	1.9	0	0.9 ± 0.1	29.7 ± 1.9	4.7 ± 1.8	0.3 ± 0.1	54.9 ± 5.2	4.9 ± 1.2	0.2 ± 0.1	42.4 ± 2.4	3.1 ± 0.5
2	4	1.9	30	9.4 ± 1.2	12.8 ± 1.7	22.3 ± 5.8	15.5 ± 2.6	39.7 ± 3.8	26.0 ± 3.3	10.8 ± 0.3	30.7 ± 0.9	23.7 ± 1.6
2	4	3.1	0	0.7 ± 0.1	37.1 ± 3.3	3.2 ± 0.9	0.3 ± 0.1	49.5 ± 5.1	1.8 ± 0.9	0.1 ± 0	42.3 ± 0.9	2.5 ± 0.4
2	4	3.1	30	5.1 ± 0.5	5.0 ± 2.3	12.7 ± 1.0	13.9 ± 2.3	27.1 ± 9.2	21.7 ± 1.1	11.8 ± 3.3	29.4 ± 5.2	23.1 ± 0.7

*Note:* Values are means ± standard error, *n* = 3. Species (Sp): 1. Tomato, 2. Cucumber; Variety (V): 1. Saluoso, 2. Sweeterno, 3. Proloog, 4. Addison; VPD: 1. 1.9 kPa, 2. 3.1 kPa; Treatment (T): 1. 0 mM, 2. 30 mM;

## Appendix 2 Mean Effects of Two Levels of Root Zone Salinity and VPD on Selected Morphological and Physiological Traits of Two Varieties of Tomato and Cucumber

**Table:**

Sp	V	VPD	T	TDW	LDW	SDW	RDW	LA	SLA	SL	R/S	SPAD
		(kPa)	(mM)	(g)	(g)	(g)	(g)	(m^2^)	(m^2^ kg^−1^)	(cm)		
1	1	1.9	0	4.3 ± 0.3	2.1 ± 0.2	1.7 ± 0.1	0.4 ± 0	0.1 ± 0	49.3 ± 1.8	72 ± 9.9	0.3 ± 0	34.6 ± 3.4
1	1	1.9	30	4.6 ± 1.3	2.4 ± 0.7	1.4 ± 0.4	0.7 ± 0.2	0.1 ± 0	65.1 ± 23.5	70. ± 2.5	0.5 ± 0	30.9 ± 1.5
1	1	3.1	0	7.1 ± 0.6	4.2 ± 0.4	1.9 ± 0.2	0.9 ± 0	0.2 ± 0	41.9 ± 3.3	70 ± 0.7	0.5 ± 0	37.6 ± 2.9
1	1	3.1	30	6.3 ± 1.1	3.5 ± 0.7	1.8 ± 0.2	1.1 ± 0.1	0.2 ± 0	51.5 ± 8.7	65.5 ± 1.8	0.6 ± 0	34.6 ± 1.0
1	2	1.9	0	4.9 ± 0.1	2.3 ± 0	1.9 ± 0.1	0.7 ± 0	0.1 ± 0	47.3 ± 4.0	68.0 ± 8.5	0.4 ± 0	35 ± 3.7
1	2	1.9	30	5.0 ± 0.8	2.7 ± 0.5	1.7 ± 0.3	0.7 ± 0.1	0.1 ± 0	49.6 ± 6.5	69.0 ± 9.8	0.4 ± 0.1	30.2 ± 2.1
1	2	3.1	0	7.3 ± 0.6	4.3 ± 0.4	2.3 ± 0.1	0.9 ± 0	0.1 ± 0	39.2 ± 0.8	77.5 ± 8.3	0.4 ± 0	36.6 ± 2.4
1	2	3.1	30	5.6 ± 0.9	3.2 ± 0.7	1.6 ± 0.1	0.8 ± 0.1	0.1 ± 0	48.3 ± 9.5	57.0 ± 2.1	0.5 ± 0	37.2 ± 2.9
2	3	1.9	0	13.4 ± 1.4	8.5 ± 1.1	3.3 ± 0.2	1.6 ± 0.1	0.4 ± 0.1	52.1 ± 1.3	115.3 ± 9.6	0.5 ± 0	35.8 ± 0.3
2	3	1.9	30	9.9 ± 2.2	6.1 ± 1.5	2.7 ± 0.6	1.1 ± 0.1	0.3 ± 0.1	56.8 ± 3.6	110.0 ± 14.2	0.4 ± 0.1	31.2 ± 2.8
2	3	3.1	0	13.8 ± 1.4	9.2 ± 1.0	3.2 ± 0.2	1.4 ± 0.2	0.5 ± 0	59.4 ± 6.4	102.6 ± 9.1	0.4 ± 0	41.7 ± 5.2
2	3	3.1	30	10.3 ± 2.0	6.7 ± 2.3	2.4 ± 0.7	1.2 ± 0.3	0.4 ± 0.1	54.5 ± 5.8	96.3 ± 23.2	0.5 ± 0.1	38.5 ± 1.0
2	4	1.9	0	15.7 ± 0.4	10.7 ± 0.3	3.4 ± 0.1	1.7 ± 0	0.7 ± 0.1	64.2 ± 8.2	104.3 ± 9.1	0.4 ± 0.1	31.8 ± 1.7
2	4	1.9	30	9.5 ± 2.0	6.3 ± 1.4	2.4 ± 0.6	0.8 ± 0	0.4 ± 0.1	62.9 ± 3.8	111.0 ± 13.5	0.4 ± 0.1	28.0 ± 0.8
2	4	3.1	0	15.5 ± 1.0	11.1 ± 0.8	3.3 ± 0.4	1.1 ± 0.1	0.7 ± 0.1	64 ± 6.1	97.5 ± 2.5	0.3 ± 0.1	38.6 ± 2.7
2	4	3.1	30	9.4 ± 2.3	6.1 ± 1.7	2.5 ± 0.5	0.9 ± 0.2	0.3 ± 0.1	56.0 ± 4.0	99.3 ± 14.2	0.4 ± 0.1	31.5 ± 0.8

*Note:* Values are means ± standard error, *n* = 3. Species (Sp): 1. Tomato, 2. Cucumber; Variety (V): 1. Saluoso, 2. Sweeterno, 3. Proloog, 4. Addison; VPD: 1. 1.9 kPa, 2. 3.1 kPa; Treatment (T): 1. 0 mM, 2. 30 mM.

Abbreviations: LA, leaf area; LDW, leaf dry weight; R/S, root/stem ratio; RDW, root dry weight; SDW, stem dry weight; SL, stem length; SLA, specific leaf area; SPAD, greenness of the leaf; TDW, total dry weight.
